# Methane Exhalation Can Monitor the Microcirculatory Changes of the Intestinal Mucosa in a Large Animal Model of Hemorrhage and Fluid Resuscitation

**DOI:** 10.3389/fmed.2020.567260

**Published:** 2020-10-22

**Authors:** Anett Bársony, Noémi Vida, Ámos Gajda, Attila Rutai, Árpád Mohácsi, Anna Szabó, Mihály Boros, Gabriella Varga, Dániel Érces

**Affiliations:** ^1^Department of Surgery, University of Szeged, Szeged, Hungary; ^2^Institute of Surgical Research, University of Szeged, Szeged, Hungary; ^3^MTA–SZTE Research Group on Photoacoustic Spectroscopy, Szeged, Hungary; ^4^Department of Optics and Quantum Electronics, Faculty of Science and Informatics, University of Szeged, Szeged, Hungary

**Keywords:** exhaled methane, diagnostic significance, small intestinal microcirculation, hemorrhage, resuscitation

## Abstract

**Background:** Internal hemorrhage is a medical emergency, which requires immediate causal therapy, but the recognition may be difficult. The reactive changes of the mesenteric circulation may be part of the earliest hemodynamic responses to bleeding. Methane is present in the luminal atmosphere; thus, we hypothesized that it can track the intestinal circulatory changes, induced by hemorrhage, non-invasively. Our goal was to validate and compare the sensitivity of this method with an established technique using sublingual microcirculatory monitoring in a large animal model of controlled, graded hemorrhage and the early phase of following fluid resuscitation.

**Materials and Methods:** The experiments were performed on anesthetized, ventilated Vietnamese minipigs (approval number: V/148/2013; *n* = 6). The animals were gradually bled seven times consecutively of 5% of their estimated blood volume (BV) each, followed by gradual fluid resuscitation with colloid (hydroxyethyl starch; 5% of the estimated BV/dose) until 80 mmHg mean arterial pressure was achieved. After each step, macrohemodynamic parameters were recorded, and exhaled methane level was monitored continuously with a custom-built photoacoustic laser-spectroscopy unit. The microcirculation of the sublingual area, ileal serosa, and mucosa was examined by intravital videomicroscopy (Cytocam-IDF, Braedius).

**Results:** Mesenteric perfusion was significantly reduced by a 5% blood loss, whereas microperfusion in the oral cavity deteriorated after a 25% loss. A statistically significant correlation was found between exhaled methane levels, superior mesenteric artery flow (r = 0.93), or microcirculatory changes in the ileal serosa (ρ = 0.78) and mucosa (*r* = 0.77). After resuscitation, the ileal mucosal microcirculation increased rapidly [De Backer score (DBS): 2.36 ± 0.42 vs. 8.6 ± 2.1 mm^−1^], whereas serosal perfusion changed gradually and with a lower amplitude (DBS: 2.51 ± 0.48 vs. 5.73 ± 0.75). Sublingual perfusion correlated with mucosal (*r* = 0.74) and serosal (*r* = 0.66) mesenteric microperfusion during the hemorrhage phase but not during the resuscitation phase.

**Conclusion:** Detection of exhaled methane levels is of diagnostic significance during experimental hemorrhage as it indicates blood loss earlier than sublingual microcirculatory changes and in the early phase of fluid resuscitation, the exhaled methane values change in association with the mesenteric perfusion and the microcirculation of the ileum.

## Introduction

The manifestation of internal bleeding varies, with the signs and symptoms usually not easily recognized; thus, a diagnosis can be difficult (1–5). Hemodynamic changes or alterations within simple laboratory parameters are often not present during the early stage of bleeding, and advanced imaging possibilities, such as CT angiography and catheter angiography, which are able to identify the presence and location of a hemorrhage, are frequently inaccessible and unsuitable for continuous monitoring (6). Nevertheless, it is recognized that the mortality rate for postoperative internal bleeding is significantly increased if higher transfusion volumes are required; therefore, the earliest possible diagnosis is necessary.

As part of the redistribution of circulation, the reduction of mesenteric perfusion is among the first homeostatic responses, and therefore a continuous, direct monitoring of blood flow in the superior mesenteric artery (SMA) and downstream intestinal microperfusion would be a highly useful, early warning tool. Today, such observations at the patient's bedside, however, are impossible. Nevertheless, non-invasive techniques with indirect monitoring options, such as sublingual capnometry and intravital microscopy methods, were developed and are in clinical use with variable success (7, 8).

We hypothesized that measurement of exhaled methane concentrations may also offer a solution to this problem. Methane in the human body originates from several sources, but it is widely accepted that it is produced by anaerobic methanogenic microorganisms, colonizing the mammalian gastrointestinal (GI) tract (9). Due to its physicochemical properties, methane is distributed evenly across membrane barriers, traverses the mucosa, and enters the mesenteric microcirculation freely (10). For continuous methane detection, near-infrared diode lasers are very effective tools for high-sensitivity photoacoustic spectroscopy (PAS) (11), and we have already provided evidence that a PAS-based breath analysis device can successfully replace gas chromatography (GC) (12).

In our earlier proof of principle study, we provided evidence that the exhaled methane levels change in association with changes in superior mesenteric arterial blood flow (13). It has been demonstrated that arterial occlusions and reperfusions and the accompanying mucosal microcirculatory cycles correlated significantly with parallel changes in methane concentration in the exhaled air (13). Therefore, the aim of the present study was to investigate the diagnostic value of real-time detection of exhaled methane levels to recognize internal bleeding in a clinically-relevant large animal model. Further aims were to examine whether continuous breath methane output monitoring can provide information on the condition of the mesenteric vascular beds during fluid resuscitation and to compare the efficacy of the technique with intravital sublingual microcirculatory analysis, a diagnostic method already in clinical use (14, 15).

## Materials and Methods

### Animals

The experiments were performed on male outbred Vietnamese minipigs (*n* = 6; 40 ± 3 kg bw) in accordance with the National Institutes of Health guidelines on the handling of and care for experimental animals and EU Directive 2010/63 on the protection of animals used for scientific purposes (approval number: V/148/2013). The animals were obtained from a local, officially licensed breeder and were kept in the animal house of the institute for an acclimatization period of 7–10 days with natural circadian light and free access to water and food. Prior to the experiments, the animals were fasted for 12 h with free access to tap water.

### Surgical Preparations

Male outbred Vietnamese minipigs (*n* = 6; weighing 40 ± 3 kg) were used. Anesthesia was induced with a mixture of tiletamine zolazepam (5 mg kg^−1^ *im*; Virbac, Carros, France) and xylazine (2 mg kg^−1^ *im*; Produlab Pharma, Raamsdonksveer, The Netherlands). The animals were placed in supine position on a warming pad with body temperature kept at 37.5 ± 0.4°C. After endotracheal intubation, mechanical ventilation was started with a tidal volume of 8–10 ml kg^−1^, and the respiratory rate was adjusted to maintain the end-tidal pressure of carbon dioxide in the 35–45 mmHg range. Anesthesia was maintained with a continuous infusion of propofol (6 mg kg^−1^ h^−1^ *iv*; Fresenius Kabi, Bad Homburg, Germany), midazolam (1.2 mg kg^−1^ h^−1^; Torrex Chiesi Pharma, Vienna, Austria), and fentanyl (0.02 mg kg^−1^ h^−1^; Richter Gedeon, Budapest, Hungary). Ringer's lactate (RL) infusion was administered at a rate of 10 ml kg^−1^ h^−1^ until bleeding was started. The depth of anesthesia was regularly controlled by monitoring the jaw tone and the absence of interdigital reflex.

The left jugular vein was cannulated with a 7F, three lumen catheter (Smiths Medical, Kirchseeon, Germany) for fluid and drug administration, as was the left femoral artery for invasive monitoring of mean arterial pressure (MAP) (PICCO Plus; PULSION Medical Systems, Feldkirchen, Germany). The left carotid artery was cannulated with a 13G single lumen catheter (Balton, Warsaw, Poland) for blood withdrawal. After median laparotomy, the SMA was dissected free, and a flow probe (Transonic Systems Inc., Ithaca, NY, USA) was placed around it to measure SMA flow. The wound in the abdominal wall had then been temporarily closed with clips until the microcirculatory investigations were started.

### Experimental Protocol

**Figure 1:**
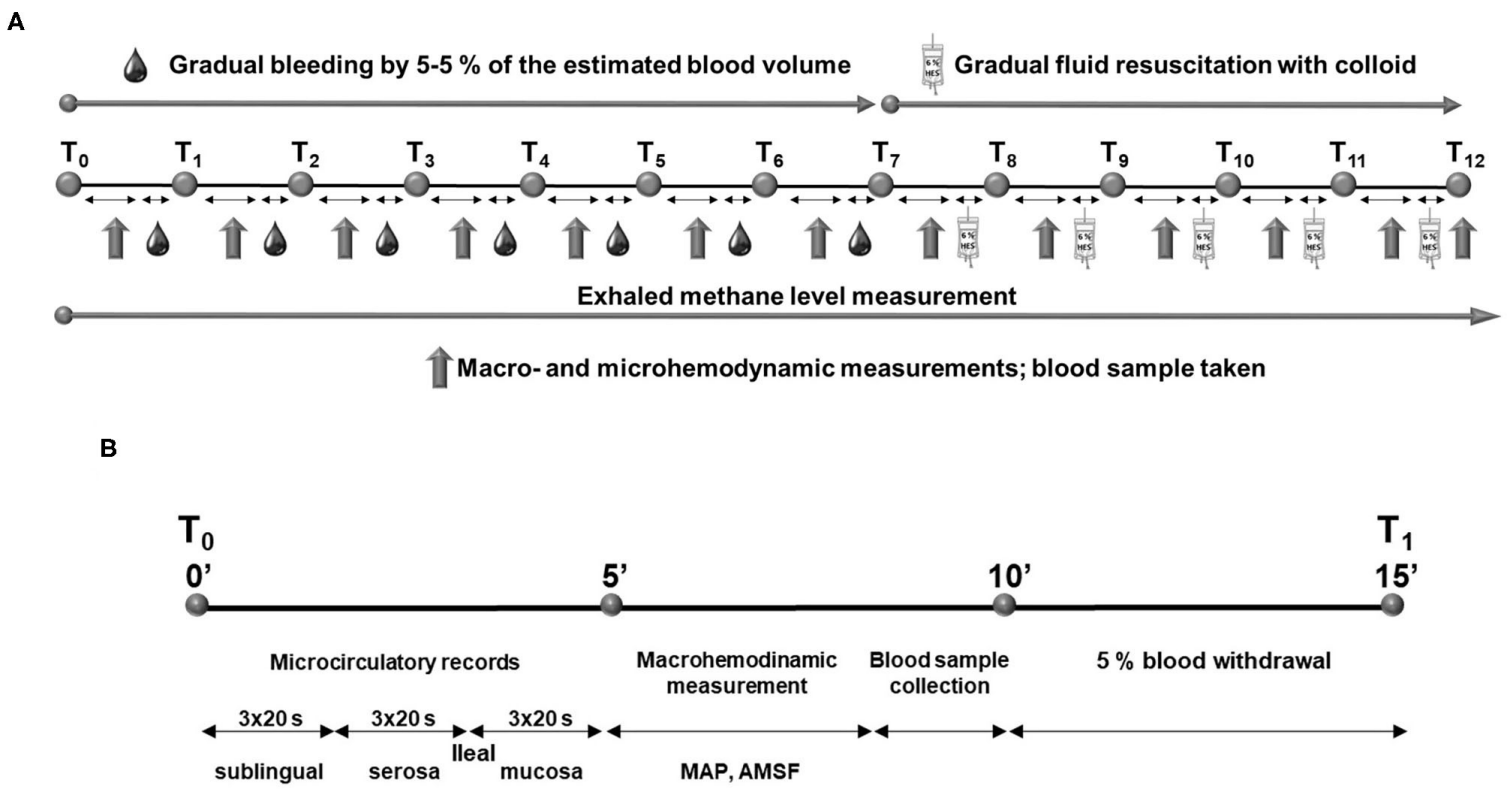
Experimental protocol **(A)** and timeline of the measurement period during the experiment **(B)**.

After the surgical preparation, a 30-min stabilizing period was provided, followed by baseline measurements. Gradual bleeding was then started. The protocol was divided into seven steps with hemorrhage (T_0_-T_6_), followed by gradual fluid resuscitation in five steps (T_7_-T_12_), until 80% of the baseline MAP value was reached (Figure 1A). The total blood volume (BV) was set as 65 ml kg^−1^, 5% of the estimated BV was withdrawn (129 ± 8 ml) by the end of each bleeding step, and an equal volume of hydroxyethyl starch (HES; Voluven 6%, 130/0.4; Fresenius Kabi, Bad Homburg, Germany) was administered during each resuscitation step.

Every bleeding or resuscitation interval was started with microcirculatory recordings at the ileal mucosal and serosal surfaces and at the sublingual area. The terminal ileum was positioned on a purpose-built investigation stand. To provide access to the ileal mucosa, a 5-cm antimesenteric incision was made with diathermy 15 cm orally from the ileo-cecal junction. The serosal and mucosal surfaces of the exteriorized ileal section were continuously rinsed with saline. At each location, three, 20-s video recordings were made. Following the intravital videomicroscopic investigations, MAP and SMA flow were recorded, and finally blood samples were taken for lactate, total hemoglobin (tHb), and hematocrit (Hct) determinations. Methane values were continuously recorded throughout the observation period. At the end of the experiments, the animals were sacrificed with an overdose of pentobarbital sodium (120 mg kg^−1^ iv; Sigma-Aldrich Inc., St. Louis, MO, USA). The timeline of measurement intervals is summarized in Figure 1B.

### Exhaled CH_4_ Analysis

We employed a near-infrared laser technique-based PS apparatus (12). PS is a subclass of optical absorption spectroscopy that measures optical absorption indirectly via the conversion of absorbed light energy into acoustic waves due to the thermal expansion of absorbing gas samples. The amplitude of the generated sound is directly proportional to the concentration of the absorbing gas component. The gas sample passes through the photoacoustic cell, in which signal generation takes place, and a microphone then detects the photoacoustic signal produced. The gas samples were taken continuously from the exhalation outlet of the ventilator at a 150 ml min^−1^ rate during the experiments. The baseline exhaled CH_4_ values were determined, and the values were thereafter subtracted from the test values. The online-detected methane values were averaged for 60-s periods to be identical with the parallel, 3 × 20-s periods of the microcirculatory analyses.

### Measurements of Lactate Level, Total Hemoglobin Concentration, and Hematocrit

Changes in tHb, Hct, and lactate concentration were analyzed with a cooximetry blood gas analyzer (Cobas b 123; Roche Ltd., Basel, Switzerland) from the arterial blood samples.

### Microcirculation Measurements

The Cytocam-Incident Dark Field (IDF) imaging technique (CytoCam Video Microscope System; Braedius Medical, Huizen, The Netherlands) was used to visualize and evaluate the microcirculation of the ileal serosal and mucosal layers and the sublingual area. IDF imaging is optimized to visualize the hemoglobin-containing structures by illuminating the organ surface with linearly polarized light (16).

Images of empty ileum segment microcirculation (serosal and mucosal surfaces) and sublingual microcirculation were recorded in three, 20-s, high-quality video clips per location by the same investigator, and records were saved as digital AVI-DV files to a hard drive. Every video clip was evaluated offline using analyzing software (AVA 3.0; Automated Vascular Analysis, Academic Medical Center, University of Amsterdam).

The capillaries (with diameter <20 μm) were categorized by sight as capillaries with no flow, sluggish flow, or continuous flow. The number of intersections of capillaries with at least sluggish flow with three equidistant horizontal and three equidistant vertical lines was counted and was manually entered in the corresponding tool in the analyzing software to calculate the De Backer score (DBS). The microvascular flow index (MFI) was determined in four quadrants of a record according to the score system defined by the MFI evaluation tool in the analyzing software: no flow (1), sluggish flow (2), or continuous flow (3). The final MFI value of a record was the average for the MFI of the four quadrants. The microvascular heterogeneity index (HI) was calculated as the difference between the highest and lowest MFIs of the three records divided by the mean MFI value of the same three videos (17). Blinded evaluation was performed by two investigators (NV and AG).

### Statistical Analysis

Data analysis was performed with a statistical software package (SigmaStat for Windows; Jandel Scientific, Erkrath, Germany). Normality of data distribution was analyzed with the Shapiro–Wilk test. The Friedman on ranks or one-way repeated measures analysis of variance (ANOVA) was applied within groups. Time-dependent differences from the baseline for each group were assessed with Dunn's method or the Bonferroni *t*-test. Differences among groups were analyzed with the Kruskal–Wallis one-way ANOVA on ranks, followed by Dunn's method. Median values and 75th and 25th percentiles are provided in the figures; *p* values < 0.05 were considered significant. Correlations between two variables were examined using Pearson's correlation coefficient (*r*) or Spearman's rank correlation coefficient (ρ); regression lines and 95% confidence intervals are provided in the figures.

## Results

### Systemic Effects of Gradual Bleeding and Resuscitation: Changes in MAP

**Figure 2:**
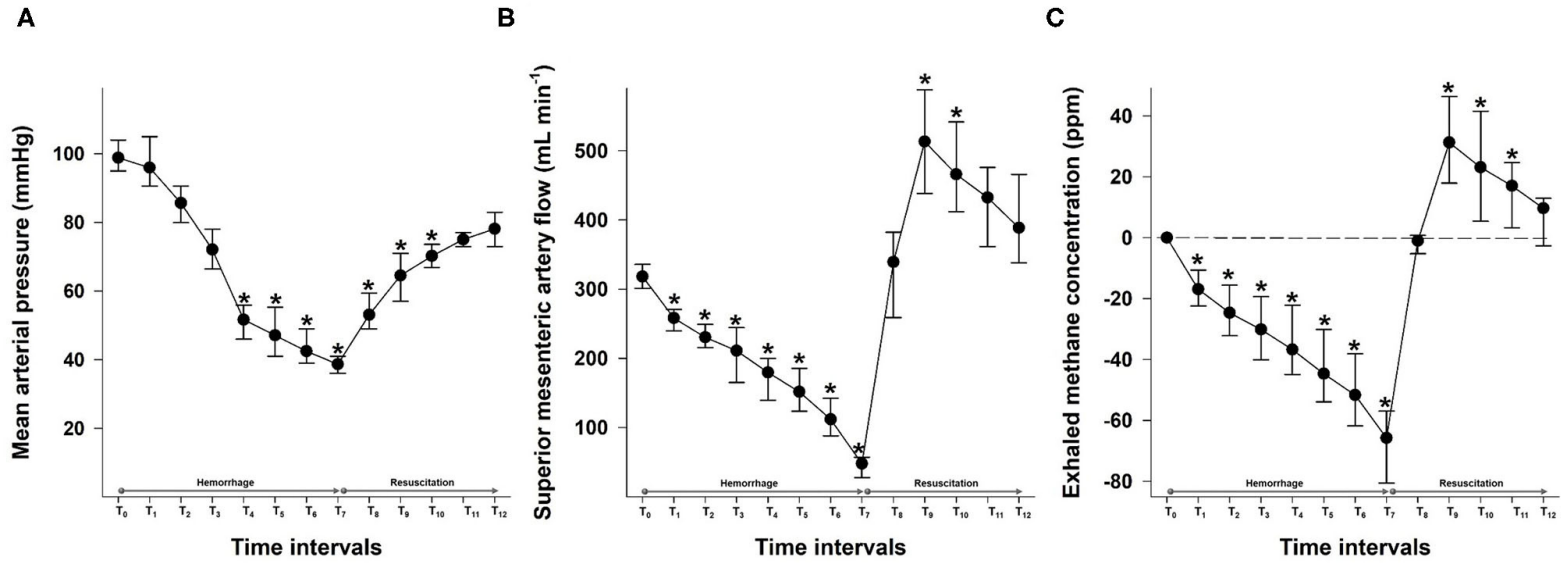
Changes in mean arterial pressure (mmHg) **(A)**, the superior mesenteric artery flow (ml min^−1^) **(B)**, and exhaled methane levels (ppm) **(C)** during the hemorrhage and resuscitation phases. The plots demonstrate the median and the 25th (lower whisker) and 75th (upper whisker) percentiles. *p < 0.05 within group vs. baseline values.

After the bleeding, MAP significantly decreased by T_3_ (20% of blood loss) and remained significantly lower until the end of the hemorrhage phase. During the resuscitation period, it remained significantly lower than the control values until T_10_, at which the volume of fluid replacement was equal to 15% of the estimated BV. By the end of the resuscitation phase, MAP reached 80% of the baseline value, as planned (Figure 2A).

The plasma lactate level was increased significantly by T_6_ (30% blood loss) and remained significantly higher than the baseline value until T_11_. The bleeding and fluid resuscitation caused a continuous decrease in both tHb and Hct values. A significant difference from the baseline values was observed at T_6_ in the case of both parameters. tHb was raised by more than 3 g dl^−1^ until the end of the hemorrhage phase (M = 11.92; p25 = 9.7; p75 = 13.4 g dl^−1^ vs. M = 8.66; p25 = 7.43; p75 = 10.48 g dl^−1^), which indicates severe bleeding (Table 1).

**Table 1:** The effects of the hemorrhage and resuscitation phases on blood lactate (mmol L^−1^), Hct (%), and tHb (g dl^−1^).

	**Parameters**	**Lactate**	**tHb**	**Hct**
**Hemorrhage phase**				
T_0_	Median	2.25	11.92	33.25
	p25; p75	1.97; 2.43	9.7; 13.4	27.75; 38.1
T_1_	Median	2.69	11.8	33.7
	p25; p75	2.2; 2.83	9.45; 12.9	28.25; 36.78
T_2_	Median	2.91	11.13	32.1
	p25; p75	2.73; 3.2	9.0; 12.4	26.9; 35.2
T_3_	Median	3.11	11.04	31.4
	p25; p75	2.77; 3.5	9.02; 12.02	25.7; 33.8
T_4_	Median	3.675	10.58	29.89
	p25; p75	3.438; 4.03	8.8; 11.45	25.5; 32.68
T_5_	Median	4.41	10.36	29.1
	p25; p75	4.27; 4.53	8.55; 11.3	24.9; 31.6
T_6_	Median	5.42^*^	10.11^*^	26.1^*^
	p25; p75	4.65; 6.05	8.28; 10.85	23.38; 29.63
**Resuscitation phase**				
T_7_	Median	6.71^*^	8.66^*^	23.68^*^
	p25; p75	5.88; 7.3	7.43; 10.48	20.9; 28.82
T_8_	Median	6.33^*^	7.99^*^	21.97^*^
	p25; p75	5.63; 7.0	7.05; 10.23	19.45; 27.97
T_9_	Median	5.4^*^	7.5^*^	20.56^*^
	p25; p75	5.13; 6.1	6.58; 9.68	18.57; 26.2
T_10_	Median	4.65^*^	7.29^*^	18.8^*^
	p25; p75	4.425; 5.4	6.6; 8.98	16.55; 24.0
T_11_	Median	4.418^*^	6.9^*^	17.38^*^
	p25; p75	3.9; 4.85	5.85; 8.2	15.43; 21.88
T_12_	Median	3.95	6.75^*^	16.37^*^
	p25; p75	3.55; 4.85	5.7; 7.87	14.4; 20.77

*The table demonstrates the median values and the 25th and 75th percentiles*.

* *p < 0.05 vs. baseline values*.

### Mesenteric Macrohemodynamics

The SMA flow decreased continuously during the hemorrhage phases. An early, significant drop was already noted at a 5% loss (T_1_) of the estimated BV. After fluid resuscitation, the MAP started to increase steeply and reached its peak value at the second resuscitation step at T_8_. During the following parts of the resuscitation phase, it decreased gradually to the level of the baseline values (Figure 2B).

### Changes in Exhaled Methane Levels

The average for baseline exhaled methane was 60.9–90.1 ppm, which corresponds to the higher range of values measured in methane-producing humans (9). The individual baseline data were subtracted from the test values to increase the comparability of measurements even in the case of larger individual variances (13).

The exhaled methane concentration decreased significantly after 5% blood loss, already at T_1_, similarly to the SMA flow changes. After resuscitation was started, breath methane level rapidly increased to a significantly higher level than the baseline and reached a peak after a fluid volume equal to 10% of the estimated BV, administered at the T_8_ period (Figure 2C).

### Changes in Sublingual and Ileal Microcirculation

**Figure 3:**
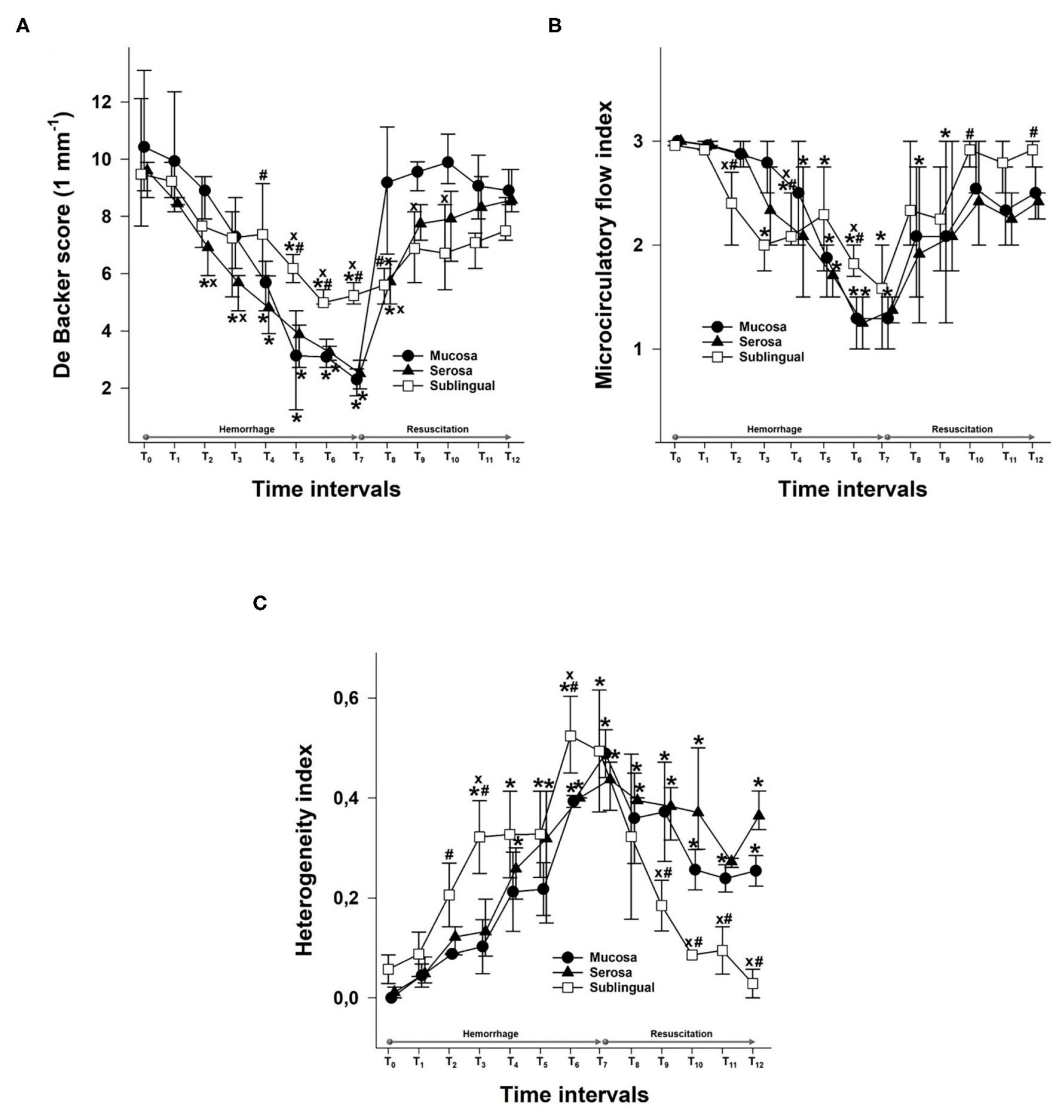
Changes in De Backer score (1 mm^−1^) values **(A)**, the microvascular flow index **(B)**, and the heterogeneity index **(C)** for the mucosa (black circles) and serosa in the ileum (black triangle) and sublingual area (empty square) during the hemorrhage and resuscitation phases. The plots demonstrate the median values and the 25th (lower whisker) and 75th (upper whisker) percentiles. ^*x*^*p* < 0.05 mucosa or sublingual values vs. serosa values; ^#^*p* < 0.05 serosa or sublingual values vs. mucosa values; *p < 0.05 vs. baseline values.

The DBS values decreased significantly as the bleeding progressed. The serosal DBS was lower than the baseline value from a 10% blood loss (T_2_). This was followed by a deterioration of mucosal DBS from the loss of 20% of the estimated BV (T_4_), whereas the decrease of DBS in the sublingual area was statistically significant from a 25% blood loss only (T_5_). Moreover, the sublingual DBS was significantly higher than the values in the ileal regions from T_5_ to T_7_, which marks the end of the hemorrhage phase and a loss of 35% of BV. When fluid resuscitation started, the mucosal DBS increased rapidly and was significantly higher than the serosal and sublingual values after fluid replacement with a volume equal to 5% of BV (T_8_), reaching the highest value at T_10_. The serosal DBS values increased more gradually with a maximum at T_12_ (Figure 3A).

Bleeding caused a decrease in the MFI in all three locations, and the first to reach significance was the MFI in the sublingual area at T_3_. This was followed by a significant decrease in serosal MFI from T_4_ and in mucosal MFI from T_5_. The fluid resuscitation resulted in a significant improvement of the MFI in all investigated locations. Sublingual MFI was significantly higher than the MFI in the ileal mucosa and serosa from T_10_ (Figure 3B).

The heterogeneity of the microcirculation increased during the hemorrhage phase as shown by the HI. The most important difference between the sublingual and ileal regions is that while the sublingual HI was restored during resuscitation, the HI in both the ileal mucosa and serosa remained significantly higher than the baseline and the sublingual values until the end of the experiments (Figure 3C).

### Link Between Changes in Exhaled Methane Concentration and Mesenteric Macro- and Microperfusion

**Figure 4:**
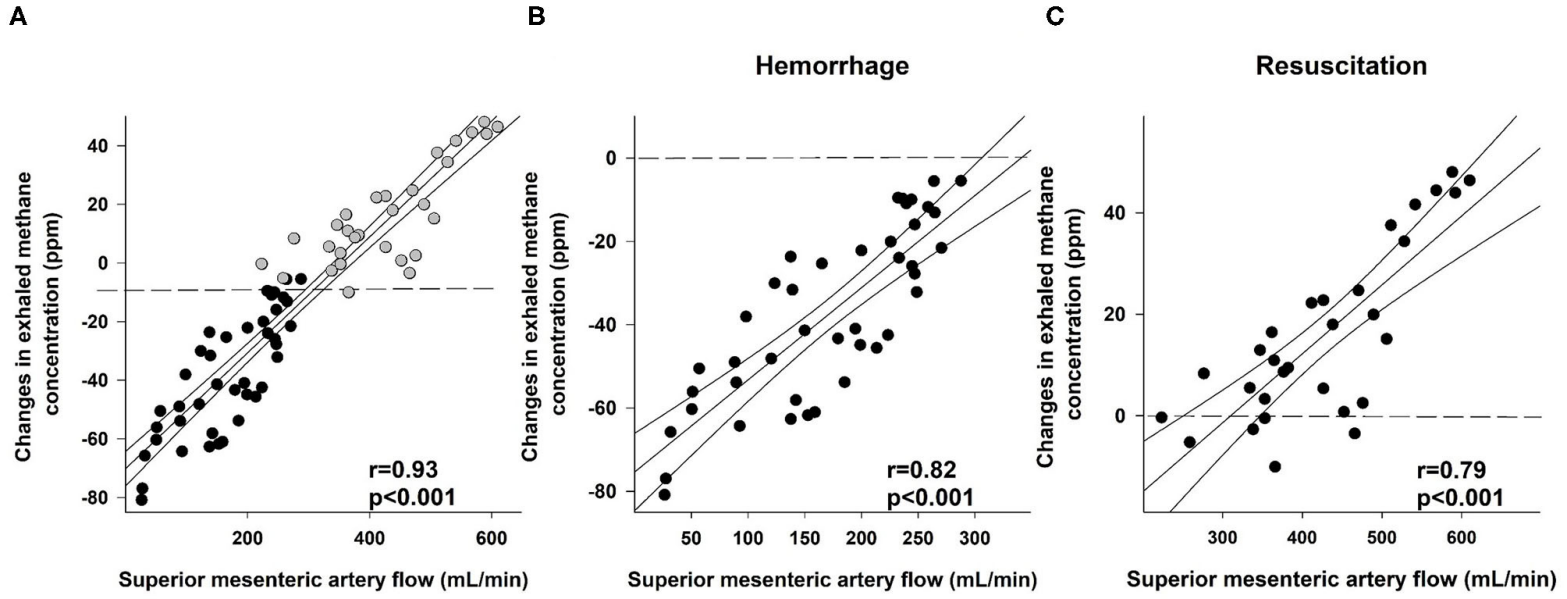
Correlation between superior mesenteric artery flow (ml min^−1^) and changes in exhaled methane concentration (ppm) during the whole course of experiments (**A**; black scatters show the data collected during bleeding, and gray scatters show the data recorded during resuscitation), the hemorrhage (**B**; black scatters) and resuscitation phases (**C**; black scatters). The plot demonstrates the regression line (black line) and corresponding *r* values as indicators of the strength of the linear correlation and *p* significance values.

**Figure 5:**
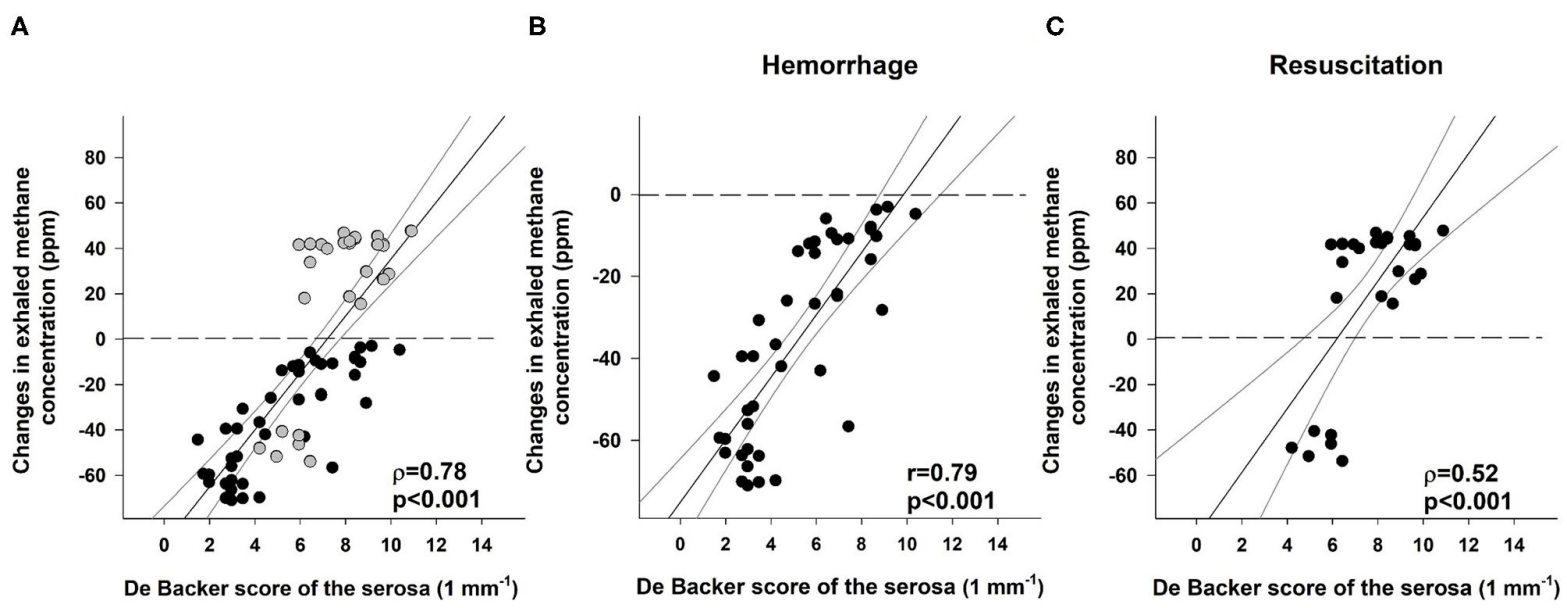
Correlation between the De Backer score (1 mm^−1^) for the serosa and changes in exhaled methane concentration (ppm) during the whole course of experiments (**A**; black scatters show the data collected during bleeding, and gray scatters show the data recorded during resuscitation), the hemorrhage (**B**; black scatters) and resuscitation phases (**C**; black scatters). The plot demonstrates the regression line (black line) and corresponding *r* and ρ values as indicators of the strength of the linear correlation and p significance values.

**Figure 6:**
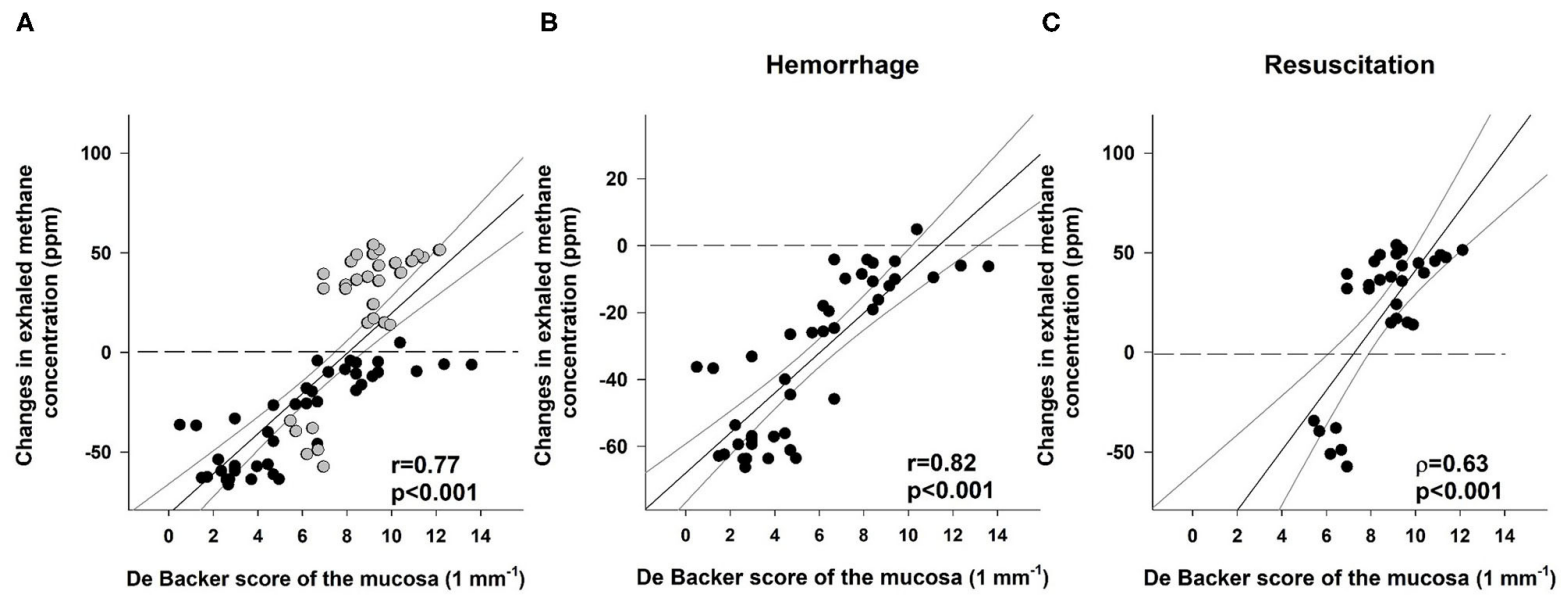
Correlation between the De Backer score (1 mm^−1^) for the mucosa and changes in exhaled methane concentration (ppm) during the whole course of experiments (**A**; black scatters show the data collected during bleeding, and gray scatters show the data recorded during resuscitation), the hemorrhage (**B**; black scatters) and resuscitation phases (**C**; black scatters). The plot demonstrates the regression line (black line) and corresponding **r** and ρ values as indicators of the strength of the linear correlation and p significance values.

**Figure 7:**
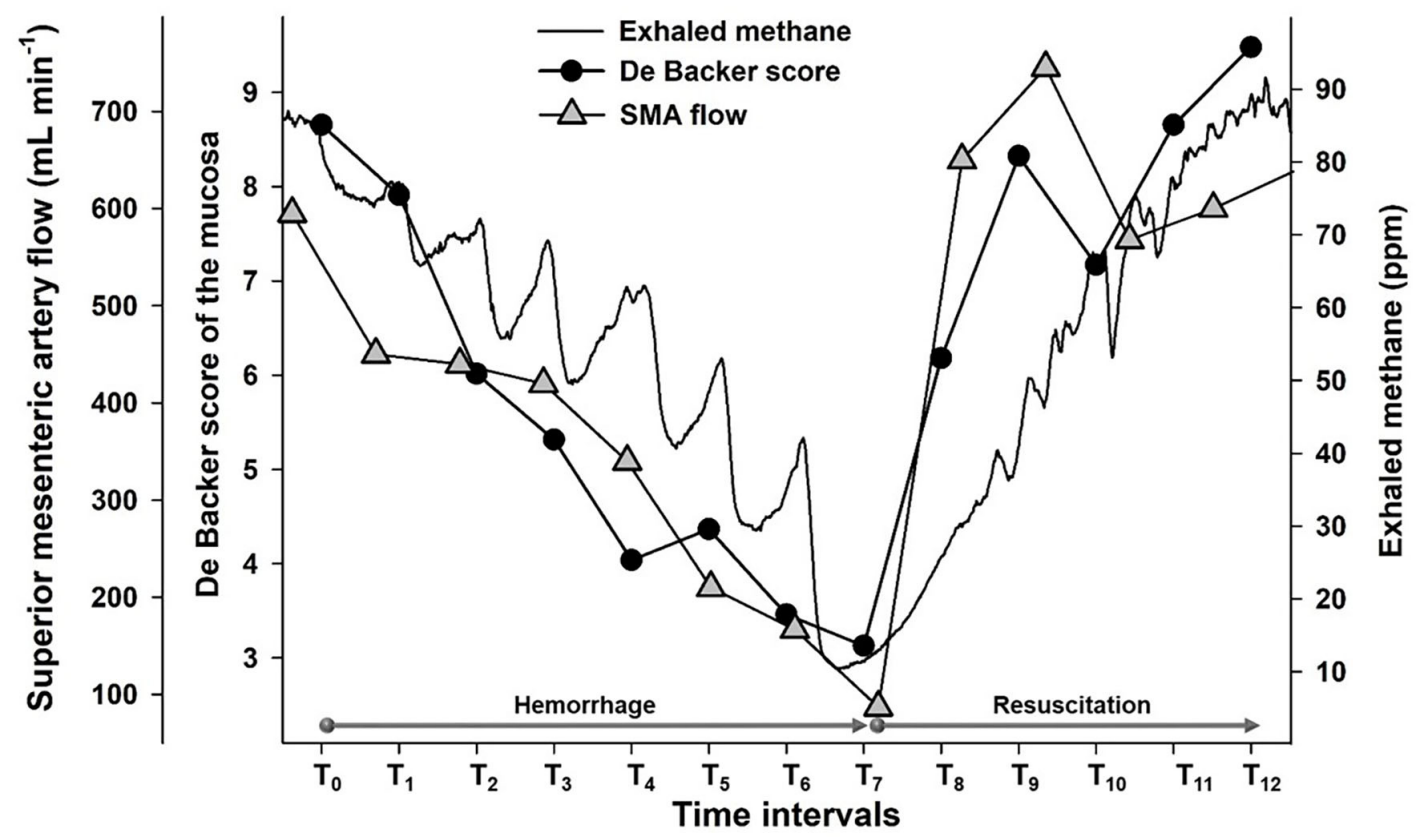
Original tracings representing changes in exhaled methane levels (ppm) (continuous black line), the De Backer score (1 mm^−1^) for ileal mucosa (black circles) and superior mesenteric artery flow (ml min^−1^) (gray triangle) of an individual animal.

We compared the changes in the exhaled methane concentration during the whole observation period with SMA flow data (*r* = 0.93; Figure 4A). Moreover, we investigated the association separately in the hemorrhage phase (*r* = 0.82; Figure 4B) and in the resuscitation phase as well (*r* = 0.79; Figure 4C). When the possible links between the changes in exhaled methane levels and the DBS values of the two components of the ileal microcirculation during the hemorrhage and resuscitation were investigated, the DBS in the serosa correlated significantly with the exhaled methane values during the experiments (ρ = 0.78; Figure 5A). When separately investigated, the correlation could be shown in both the bleeding phase (*r* = 0.79; Figure 5B) and the fluid resuscitation period (ρ = 0.52; Figure 5C). Similarly, a significant correlation was present in the case of the mucosal DBS values when the whole data set was analyzed (*r* = 0.77; Figure 6A) and also when data were separated to the hemorrhage (*r* = 0.82; Figure 6B) and resuscitation phases (ρ = 0.63; Figure 6C). Phases are shown to demonstrate the changes in exhaled methane concentrations and the DBS of the ileal mucosa on an original methane registration curve of a single animal and the simultaneous changes in the SMA flow and mucosal DBS in the same animal during hemorrhage and resuscitation (Figure 7).

### Correlations Between Sublingual and Ileal Mucosal or Serosal Microcirculation

**Figure 8:**
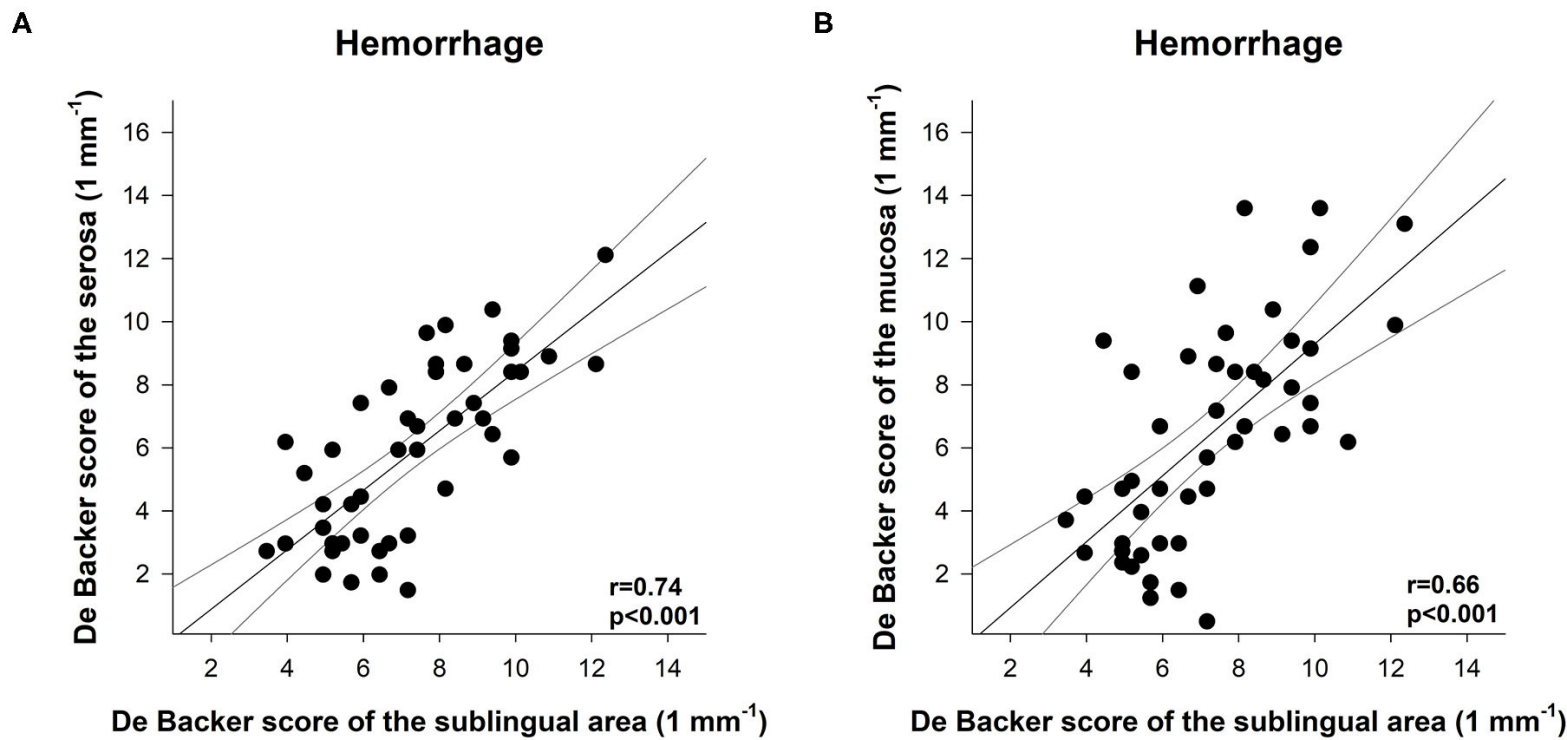
Correlation between the De Backer score (1 mm^−1^) for the serosa **(A)** or mucosa **(B)** and the De Backer score (1 mm^−1^) for the sublingual area during the hemorrhage phase. The plot demonstrates the regression line (gray line) and corresponding (black scatters) *r* values as an indicator of the strength of the linear correlation and *p* significance values.

Significant correlations were detected during the hemorrhage phase between the sublingual DBS and the serosal or mucosal DBS values (*r* = 0.74 and *r* = 0.66, respectively; Figures 8A,B).

## Discussion

We used continuous, real-time detection of exhaled methane concentration to investigate the link to the macro- and microvascular components of the mesenteric circulation during and after hemorrhage. The changes in SMA flow developed earlier than systemic hemodynamic or Hct responses, and the changes in exhaled methane levels strictly followed the mesenteric alterations. Therefore, we propose that monitoring methane in the exhaled air may be an early warning tool to recognize internal hemorrhage. Moreover, exhaled methane monitoring can provide information to estimate the condition of the microcirculatory part of the mesenteric region during bleeding, and it is capable of following the sudden changes during the very early phase of fluid resuscitation. Nonetheless, we are aware that an important limitation is the detection of baseline values in certain clinical situations. Here, it should be noted that real-time breath methane monitoring technique may increase the diagnostic potential of previous, traditional methods. This approach is based on dynamic, constant tracking instead of detection static values in a given time. Any observation (increase or decrease) can direct the attention toward a possible disturbance of the mesenteric circulation.

The methane breath test is already used to diagnose certain GI disorders. In human clinical laboratory practice, breath methane levels are usually determined by a lactulose test and sampling of breath air in gas-tight bags, which are then analyzed by GC, equipped with either flame ionization, thermal conductivity, or mass spectrometry detectors (18). Here, it should be noted that the sampling frequency of these traditional methods is limited. Our approach is somehow different; in contrast to the GC technique, PAS provides the option to follow real-time changes at a sensitivity threshold < 1 ppm compared with the 3 ppm sensitivity threshold of the presently available GC instruments. With this method, the dynamics of exhaled methane concentrations can also be followed in single breath sample analyses (12).

We used an anesthetized, acute pig model for a gradual, relatively low rate (5% loss of BV in each step), but severe hemorrhage was followed by a controlled, gradual and restricted (80% of the baseline MAP) fluid resuscitation. The total BV loss was set at 35%, which resulted in an approximately 3 g dl^−1^ loss of tHB, confirming the severity of the bleeding. The low rate provided a good temporal resolution with the possibility of seven measurement intervals during the hemorrhage phase and five intervals until the goal MAP was reached in the resuscitation phase. We decided to use HES as resuscitation fluid, as it was expected to provide pronounced macrohemodynamic changes and was also capable of restoring intestinal microcirculation (19).

As expected, the SMA blood flow was affected very early, already significantly decreased after 5% blood was withdrawn. Changes in the exhaled methane concentration followed the decrease with the same dynamics. Significant changes in the DBS of the serosal and mucosal components of the ileal microcirculation occurred slightly later, after a 10 or 20% blood loss, respectively. The difference between the mesenteric macro- and microcirculation might be explained by a possible autoregulation of mesenteric microperfusion (20), whereas the delay between the mucosal and serosal microcirculatory changes may be a result of the phenomenon of microcirculatory redistribution, which supports the oxygenation of the mucosa at the price of reduced serosal perfusion (21). The earlier decrease of the exhaled methane level might indicate a reduced absolute volume of the perfused blood, without the decrease of perfused capillary density.

At the beginning of the resuscitation phase (T_7_-T_8_), a sudden increase in the SMA flow and mucosal DBS and a rise in exhaled methane levels were observed. However, by the end of the experiments, the exhaled methane and SMA flow values decreased, and no significant difference could be detected compared with the baseline values. The DBS of both the mucosal and serosal areas remained steady during the whole resuscitation phase, and no decrease was observed in the last two resuscitation intervals. Nonetheless, the HI was increased by the end of the hemorrhage phase and remained elevated during the entire resuscitation phase, which suggests that the microcirculation was not completely restored by the fluid replacement and might explain the methane decrease after the initial peak.

The microcirculation of the sublingual area is frequently investigated, as it is considered a suitable GI region for non-invasive approaches, assuming that the changes might indicate the condition of the microcirculation in more distal sections, such as the ileum. Indeed, earlier studies demonstrated that tissue carbon dioxide pressure in the sublingual area is tied to changes in the small intestinal microcirculation in a hemorrhagic shock and fluid resuscitation model (22). In the present study, we could not detect a correlation between the sublingual microcirculation and the serosal or mucosal components of ileal microperfusion in the resuscitation phase, and this finding highlights the difference between the two methods. An investigation of the sublingual area is capable of following the GI microcirculatory changes in a wider timeframe only, and this is not affected by an increase in the sampling frequency because of the inertia between the sublingual and more distal microcirculatory regions. On the other hand, the dynamics of the changes in the exhaled methane concentrations were similar to those of the changes in the mesenteric circulation. Real-time monitoring of exhaled methane level was capable of following the sudden changes observed at the very early resuscitation phase.

However, the changes of methane levels increased with a slightly lower rate following the microcirculatory changes during the resuscitation phase than in the hemorrhage period. The lower correlation coefficients between exhaled methane levels and serosal or mucosal DBS values also refer to the role of the characteristics of the changes of the mesenteric perfusion. The different kinetics of the relationship of exhaled methane levels and microcirculatory changes might be explained by the rapid improvement of the microperfusion at the early phase of resuscitation. We suggest a two-compartment model, in which the lumen and the wall of the intestines is one of the components, and the circulating blood is the other part. In this case, the disproportionate increase in the blood flow and methane access to the circulatory compartment may explain the lower than expected exhaled methane output during the resuscitation phase.

In conclusion, changes in exhaled methane concentration may indicate bleeding at an early stage and follow changes in mesenteric perfusion during hemorrhage and resuscitation as well, with a diagnostic value comparable to the monitoring of the sublingual microcirculatory area. It might be a useful, additional non-invasive tool in cases where hemorrhagic complications might be expected; however, even in its current form, the technique might contribute to acquiring additional information on the mesenteric circulation in experimental setups.

## Data Availability Statement

The raw data supporting the conclusions of this article will be made available by the authors, without undue reservation.

## Ethics Statement

The animal study was reviewed and approved by National Scientific Ethical Committee on Animal Experimentation (National Competent Authority of Hungary).

## Author Contributions

AB, GV, MB, and DÉ performed experiments and wrote the manuscript. AR performed experiments and figures. NV and ÁG prepared figures and evaluated microcirculatory records. GV, DÉ, and MB supervised and edited the manuscript. AS and ÁM designed and constructed the photoacoustic spectroscopy device and supervised the manuscript. All authors contributed to the article and approved the submitted version.

## Conflict of Interest

The authors declare that the research was conducted in the absence of any commercial or financial relationships that could be construed as a potential conflict of interest.
